# Development and validation of a prognostic model of resectable small-cell lung cancer: a large population-based cohort study and external validation

**DOI:** 10.1186/s12967-020-02412-x

**Published:** 2020-06-15

**Authors:** Yu Wang, Zhaofei Pang, Xiaowei Chen, Tao Yan, Jichang Liu, Jiajun Du

**Affiliations:** 1 Institute of Oncology, Shandong Provincial Hospital, Affiliated to Shandong First Medical University, 324 Jingwu Road, Jinan, 250021 Shandong People’s Republic of China; 2 Department of Oncology, Shandong Provincial Hospital, Affiliated to Shandong First Medical University, 324 Jingwu Road, Jinan, 250021 Shandong China; 3 Department of Thoracic Surgery, Shandong Provincial Hospital, Affiliated to Shandong First Medical University, 324 Jingwu Road, Jinan, 250021 Shandong China

**Keywords:** Small cell lung cancer, Resectable, Nomogram, Surgery

## Abstract

**Background:**

Survival outcomes of patients with resected SCLC differ widely. The aim of our study was to build a model for individualized risk assessment and accurate prediction of overall survival (OS) in resectable SCLC patients.

**Methods:**

We collected 1052 patients with resected SCLC from the Surveillance, Epidemiology, and End Results (SEER) database. Independent prognostic factors were selected by COX regression analyses, based on which a nomogram was constructed by R code. External validation were performed in 114 patients from Shandong Provincial Hospital. We conducted comparison between the new model and the AJCC staging system. Kaplan–Meier survival analyses were applied to test the application of the risk stratification system.

**Results:**

Sex, age, T stage, N stage, LNR, surgery and chemotherapy were identified to be independent predictors of OS, according which a nomogram was built. Concordance index (C-index) of the training cohort were 0.721, 0.708, 0.726 for 1-, 3- and 5-year OS, respectively. And that in the validation cohort were 0.819, 0.656, 0.708, respectively. Calibration curves also showed great prediction accuracy. In comparison with 8th AJCC staging system, improved net benefits in decision curve analyses (DCA) and evaluated integrated discrimination improvement (IDI) were obtained. The risk stratification system can significantly distinguish the ones with different survival risk. We implemented the nomogram in a user-friendly webserver.

**Conclusions:**

We built a novel nomogram and risk stratification system integrating clinicopathological characteristics and surgical procedure for resectable SCLC. The model showed superior prediction ability for resectable SCLC.

## Background

Worldwide, lung cancer remains an important public health concern affecting both men and women and the leading cause of cancer-associated mortality [1]. In the United states, there were estimated 234,030 new diagnosed lung cancer cases in 2018 [1]. Small-cell lung cancer (SCLC) is one of the easily aggressive pathology type and accounts for approximately 14–16% of all lung cancer cases [2, 3].

SCLC is the mainly neuroendocrine tumor of lung which has poor prognosis for its high vascularity, rapid doubling time and early metastasis. Mainly treating choices of SCLC include surgery, chemotherapy and radiotherapy [4]. Systemic platinum-based chemotherapy either alone or combined with concurrent radiotherapy is most commonly considered to be standard and potentially curative treatment for SCLC lesions, because most SCLC cases are highly sensitive to initial chemotherapy and radiotherapy [5]. But patients often develop treatment-resistance quickly and subsequent relapse and eventual death.

The role of surgery in SCLC was reevaluate over and over again. Before 1970s, surgery was a common treating modality for SCLC, which was overturned by a Medical Research Council trial performed in 1973. This trial demonstrated the poor survival of SCLC patients with pulmonary resection than radiotherapy [6]. Besides, the results of another prospective randomized trial in 1994 did not support the addition of pulmonary resection to the multimodality treatment of small cell lung cancer [7]. These vital evidences led to abandonment of surgery as a standard treatment. But renewed studies advocated adopting surgery to increase local-control rate in certain early-stage SCLC. A study published in 2010 re-evaluated the role of surgery, and showed that lobectomy, in selected patients with limited-SCLC was associated with improved survival outcomes [8]. And the research which focused on survival of patients with SCLC undergoing lung resection in 1998–2009 in England also suggested surgical resection for early stage SCLC [9]. A Italian review published in 2015 summarized recent original researches and suggested that surgery should be offered (or at least considered) in intraoperative diagnosis of resectable SCLC or early-stage SCLC after chemotherapy [10]. Therefore, more reasonable or proper staging and prognostic prediction is extremely important for surgical procedure and even following survival outcomes.

Most clinical guidelines for SCLC were based on the VALSG staging system in which SCLC patients were roughly distributed into extensive-stage and limited-stage. However, it has been recommended that the AJCC TNM staging system should replace the VALSG staging system because TNM system would allow for more proper treating selections (e.g. surgical resection) and more precise prognostic assessments [5]. Nevertheless, except for TNM staging status, it was known that clinical characteristics like sex, age, location and treating modalities were also noteworthy factors influencing individual survival outcomes of cancer patients [10–12]. For instance, lobectomy was demonstrated to have superior survival outcomes compared with sublobectomy or pneumonectomy [13–15]. Above all, it is obvious that the TNM system is less sufficient for predicting outcomes of an individualized resectable SCLC patient. Therefore, a more refined model with better prognostic discrimination of is required, and a nomogram is an ideal tool to solve this problem [16, 17].

Nomogram is a tool to predict individual prognosis of patients by regression analyses of the potential prognostic factors. Previous four nomograms were built by different institutions involving SCLC patients, but there still lack efficient nomogram that can predict survival outcomes of resectable SCLC patients specially [18–21]. The objective of this study was to derive and externally validate a prognostic nomogram to predict overall survival (OS) for patients who did resection of SCLC in two independent cohorts, which would help clinical decision making and to assist ongoing efforts.

## Materials and methods

### Training cohort and data

The flow chart of this study was shown in Additional file 1.

The data of patients with SCLC diagnosed from 2004 to 2016 were retrieved from the SEER 18 database using the SEER*Stat program (v 8.3.5). The SEER program is a public national database which contains data on cancer occurrences in 18 areas of United States and covers approximately 26% of the population. Among these patients, there were 1485 patients conformed to our inclusion criteria: only one primary tumor; diagnosis confirmed by histology; histological type of small-cell carcinoma (ICD-O-3); surgery performed. SCLC, also named oat cell carcinoma, with histological codes included as follows: 8041/3, 8042/3, 8043/3, 8044/3, 8045/3. Variables with more than 10% missing values (Blanks or unknown or N/A are deemed as missing) were not eligible for analysis. Eventually, 1052 patients were included for analyses, after excluding the following ineligible cases: 411 patients with 8th TNM stage of M1/N3/Tx/Nx/Mx, 22 patients with unknown surgery details, 4 patients with no access to data of lymph nodes metastatic ratio (LNR). There were 4 patients that meet more than one of above exclusion criteria. LNR was the number of lymph nodes with metastasis divided by the total number of dissected lymph nodes [22].

The data included clinical information of patients, histological characteristics, survival time (months) and vital status (the event of death). Continuous variables were transformed into categorical variables based on recognized cutoff values (for age). Clinical information of patients included sex (female v male), age (≤ 60 years, 60–70 years, > 70 years), marital status (unmarried, married), surgery (lobectomy, sublobectomy, pneumonectomy). Pathological characteristics of tumors include primary site (upper lobe, lower lobe, other), lateral (left, right), pathological grade (I–II, III, IV), T stage in 8th edition AJCC system (T1, T2, T3–T4), N stage in 8th edition AJCC system (N0, N1, N2), LNR (< 0.01, > 0.01, no resected lymph node), radiotherapy or not, chemotherapy or not. The acquisition of cutoff value of LNR was achieved by the receiver operating characteristic (ROC) analysis. The time of last follow-up was December 2016. The primary outcome was defined as overall survival (OS). Time of OS was counted from date of diagnosis to date of death or last contact.

### External validation cohort and data

To further validate our new model in a responsible manner, we sought an external validation cohort from patients diagnosed from January 2004 to December 2016 in Shandong Provincial Hospital Affiliated to Shandong University, Shandong Provincial Hospital Affiliated to Shandong First Medical University. The validation cohort included 114 postoperative SCLC patients who were recruited according to inclusion and exclusion criteria same as the training cohort. We collected variables according the training cohort except for marital status and pathological grade. The time of last follow-up was July 2019. The outcome variable was OS too.

The ethical committee and institutional review board of Shandong Provincial Hospital c approved this study.

### Construction and evaluation of the prognostic model

To identify independent prognostic factors to build our prognosis model, we performed univariate COX Proportional Hazard Regression analysis in a forward stepwise manner. Significant factors in univariate analysis (p value < 0.05) were carried into a multivariate COX Proportional Hazard Regression analysis to obtain the hazard ratio (HR) and corresponding 95% confidential interval (CI) for every independent prognostic variable. All the COX Regression analyses were performed by SPSS 25.0 (SPSS, Chicago, IL). The prognostic nomogram was built based on surgery methods and other independent prognostic variables by using the survival and rms packages of R 3.5.1.

Evaluation of a nomogram generally include two facets: discrimination and calibration accuracy. Discrimination means the efficiency of the model to distinguish patients with different survival outcomes. Usually, concordance index (C-index) is taken to be the tool to measure discrimination, which represents a concordance measure analogous to area under the receiver operating characteristic (ROC). Values of C-index range from 0.5 (no discrimination) to 1.0 (perfect discrimination). Calibration accuracy measures how the predicted probabilities are close to actual survival outcomes which is showed in the form of calibration curves. Calibration curves of the nomogram for 1-, 3- and 5-year OS were achieved by use of the “survival and rms” R package in the training and validation cohort. All the evaluation processes were performed by bootstrapping for 1000 times.

To further evaluate the benefits and advantages of our new predicting model, we adopted decision curve analysis (DCA). DCA is usually used to evaluate alternative diagnostic and prediction strategies that have advantages over other generally used measures and techniques. If the threshold probability of net benefits of the new prediction model is unpractical, the benefits of it will be less than the benefits of existing tools (for example, 8th edition AJCC TNM staging system), which means poor applicability. Integrated discrimination improvement (IDI) index were employed to assess whether the new model more accurate than 8th edition AJCC TNM staging system or not. A larger difference value between the death probability of individual patient predicted by the new model and TNM staging system demonstrates a better predicting accuracy. Z test was performed to examine the significance of IDI between the new model and the 8th edition AJCC staging.

### Construction of a risk stratification model

Based on the aggregate score of every patient on the nomogram, the cohort was distributed into two different risk groups (low and high). We obtained the most propriate cutoff value by use of receiver operating characteristic (ROC) analysis. To test the application of the risk stratification model, we conducted Kaplan–Meier survival analyses in both the training cohort and the validation cohort with Chi square test. A two-sided P value < 0.05 was deemed significant.

RStudio software (version 1.1.463) was used to perform the survival and RMS R package. Details of all R code involving generation and further evaluations of the model were shown in Additional file 2. This study followed the TRIPOD (Transparent Reporting of a multivariable prediction model for Individual Prognosis Or Diagnosis) statement (Additional file 3) and adhered to the Declaration of Helsinki for medical research involving human subjects.

### Creation of a webserver for the nomogram

To facilitate clinicians’ usage of our nomogram, we created a user-friendly webserver. The webserver can calculate a survival probability as long as you input correct information of a SCLC with surgery performed and certain prediction time (months) such as 12 months. Meanwhile, it can also provide the corresponding survival plot of this case.

## Results

### Clinicopathological characteristics of the study cohorts

Eventually, after the stepwise selection, a total of 1052 cases from SEER database were included into the training cohort, and 114 cases from Shandong Provincial Hospital were involved into the external validation cohort. The clinical characteristics of patients and pathological characteristic of tumors were summarized in Table 1. In the training cohort, 634 (60.3%) underwent lobectomy, 382 (36.3%) performed sublobectomy, 36 (3.4%) did pneumonectomy. In the external validation cohort, 78 (68.4%) underwent lobectomy, 9 (7.9%) accepted sublobectomy, 27 (23.7%) received pneumonectomy. Patients with larger tumor size (T stage) were more likely to receive pneumonectomy. Less than half of the cases received radiotherapy as adjuvant therapy (39.2% and 27.2% in the training and validation cohort, respectively). The proportion of radiotherapy-received patients was larger in sublobectomy-subgroup than other surgical procedure subgroups (46.1% of the training cohort and 44.4% of the validation cohort). Over half of the patients received chemotherapy (68.5% of the training cohort and 73.7% of the validation cohort). Besides, the proportion was much higher in sublobectomy subgroup.

**Table 1:** Clinical and tumor characteristics of training and validation cohort

Variables	Training cohort (n = 1052)			Validation cohort (n = 114)		
	No. (%)			No. (%)		
	Lobe-	Sublob-	Pneumo-	Lobe-	Sublob-	Pneumo-
Sex						
Male	282 (44.5)	167 (43. 7)	15 (41.7)	52 (66.7)	7 (77.8)	19 (70.4)
Female	352 (55.5)	215 (56.3)	21 (58.3)	26 (33.3)	2 (22.2)	8 (29.6)
Age						
≤ 60	169 (26.7)	99 (25.9)	16 (44.5)	41 (52.5)	5 (55.6)	22 (81.5)
60–70	257 (40.5)	130 (34.0)	12 (33.3)	30 (38.5)	3 (33.3)	5 (18.5)
> 70	208 (32.8)	153 (40.1)	8 (22.2)	7 (9.0)	1 (11.1)	0 (0.0)
Marital						
Unmarried	287 (45.3)	167 (43.7)	11 (30.6)	–	–	–
Married	347 (54.7)	215 (56.3)	25 (69.4)	–	–	–
Lateral						
Left	282 (44.5)	175 (45.8)	23 (63.9)	38 (48.7)	3 (33.3)	22 (81.5)
Right	352 (55.5)	207 (54.2)	13 (36.1)	40 (51.3)	6 (66.7)	5 (18.5)
Primary site						
Upper lobe	377 (59.5)	235 (61.5)	18 (50.0)	32 (41.0)	6 (66.7)	6 (22.2)
Lower lobe	202 (31.8)	90 (23.6)	7 (19.4)	34 (43.6)	2 (22.2)	6 (22.2)
Other	55 (8.7)	57 (14.9)	11 (30.6)	12 (15.4)	1 (11.1)	15 (55.6)
Grade						
I–II	30 (4.7)	14 (3.7)	4 (11.1)	–	–	–
III	227 (35.8)	107 (28.0)	9 (25.0)	–	–	–
IV	191 (30.1)	97 (25.4)	13 (36.1)	–	–	–
Unknown	186 (29.4)	164 (42.9)	10 (27.8)	–	–	–
T stage						
T1	326 (51.4)	197 (51.6)	5 (13.9)	26 (33.3)	3 (33.3)	1 (3.7)
T2	231 (36.4)	107 (28.0)	10 (27.8)	29 (37.2)	4 (44.5)	6 (22.2)
T3–T4	77 (12.2)	78 (20.4)	21 (58.3)	23 (29.5)	2 (22.2)	20 (74.1)
N stage						
N0	387 (61.0)	229 (59.9)	11 (30.6)	25 (32.1)	3 (33.3)	4 (14.8)
N1	142 (22.4)	43 (11.3)	15 (41.7)	21 (26.9)	3 (33.3)	7 (25.9)
N2	105 (16.6)	110 (28.8)	10 (27.8)	32 (41.0)	3 (33.3)	16 (59.3)
LNR						
< 0.01	379 (59.8)	125 (32.7)	11 (30.6)	23 (29.5)	2 (22.2)	3 (11.1)
> 0.01	235 (37.0)	98 (25.7)	21 (58.3)	54 (69.2)	6 (66.7)	23 (85.2)
NO*	20 (3.2)	159 (41.6)	4 (11.1)	1 (1.3)	1 (11.1)	1 (3.7)
Radiation						
No	412 (65.0)	206 (53.9)	22 (61.1)	55 (70.5)	5 (55.6)	23 (85.2)
Yes	222 (35.0)	176 (46.1)	14 (38.9)	23 (29.5)	4 (44.4)	4 (14.8)
Chemo						
No	201 (31.7)	117 (30.6)	13 (36.1)	18 (23.1)	2 (22.2)	10 (37.0)
Yes	433 (62.3)	265 (69.4)	23 (63.9)	60 (76.9)	7 (77.8)	17 (63.0)

*LNR* lymph node metastatic ratio, *Lobe-* lobectomy, *Sublob-* sublobectomy, *Pneumo-* pneumonectomy, NO* no lymph node resected; *Chemo* chemotherapy

### Risk factors for overall survival

There were 650 events (deaths) in the training cohort and the mean follow-up period was 34.58 months (median, 21 months; range, 0–155 months).

In the univariate COX Regression analyses, sex, age, T stage, N stage, LNR, surgery and chemotherapy were significantly associated with overall survival (Table 2). However, marriage, tumor locations, histological grade and radiotherapy didn’t show significance to survival. All the seven significant factors eventually incorporated into the multivariate COX Regression analysis were demonstrated to be independent prognostic factors (Table 2). Male, age > 70 years, higher T or N stage, no resected lymph node, pneumonectomy and no chemotherapy were proved to have higher hazard of death. In terms of surgical procedure, lobectomy was associated with the lowest risk of death (sublobectomy vs lobectomy, HR: 1.444; p < 0.001; pneumonectomy vs lobectomy, HR: 1.556; p = 0.024).

**Table 2:** Cox regression analyses of prognostic variables for OS

Variables	Univariate cox regression		Multivariate cox regression	
	HR (95% CI)	p value	HR (95% CI)	p value
Sex				
Male	1.193 (1.023–1.393)	0.025	1.209 (1.035–1.413)	0.017
Female	1		1	
Age		< 0.001		< 0.001
≤ 60	0.550 (0.449–0.673)	< 0.001	0.492 (0.400–0.605)	< 0.001
> 60, ≤ 70	0.699 (0.586–0.833)	< 0.001	0.747 (0.624–0.895)	0.002
> 70	1		1	
Marital status				
Unmarried	1.000 (0.856–1.168)	0.998	–	–
Married	1		–	–
Lateral			–	–
Left	1.019 (0.873–1.189)	0.810	–	–
Right	1		–	–
Primary site		0.282		–
Upper lobe	1		–	
Lower lobe	1.054 (0.885–1.256)	0.556	–	–
Other	1.205 (0.956–1.519)	0.114	–	–
Grade		0.095	–	–
I–II	0.625 (0.414–0.945)	0.026	–	–
III	0.875 (0.726–1.055)	0.163	–	–
IV	0.865 (0.713–1.049)	0.140	–	–
Unknown	1		–	–
T stage		< 0.001		< 0.001
T1	1		1	
T2	1.267 (1.062–1.511)	0.009	1.253 (1.046–1.501)	0.015
T3–T4	2.106 (1.725–2.572)	< 0.001	2.000 (1.622–2.467)	< 0.001
Surgery		< 0.001		< 0.001
Lobectomy	1		1	< 0.001
Sublobectomy	1.659 (1.415–1.944)	< 0.001	1.444 (1.194–1.747)	< 0.001
Pneumonectomy	2.006 (1.381–2.915)	< 0.001	1.556 (1.060–2.285)	0.024
N stage		< 0.001		0.022
N0	1		1	
N1	1.570 (1.292–1.908)	< 0.001	1.529 (1.064–2.196)	0.022
N2	1.865 (1.548–2.246)	< 0.001	1.572 (1.139–2.170)	0.006
LNR		< 0.001		0.005
< 0.01	1		1	
> 0.01	1.894 (1.590–2.256)	< 0.001	1.310 (0.921–1.864)	0.1330
NO*	2.177 (1.771–2.676)	< 0.001	1.537 (1.186–1.992)	0.001
Radiation				–
No	1		–	–
Yes	1.008 (0.862–1.179)	0.919	–	–
Chemotherapy				
No	1.254 (1.064–1.476)	0.007	1.525 (1.282–1.815)	< 0.001
Yes	1		1	

*CI* confidence interval, *HR* hazard ratio, *OS* overall survival, *LNR* lymph node metastatic ratio, NO* no lymph node resected

### Prognostic nomogram for OS

**Fig. 1:**
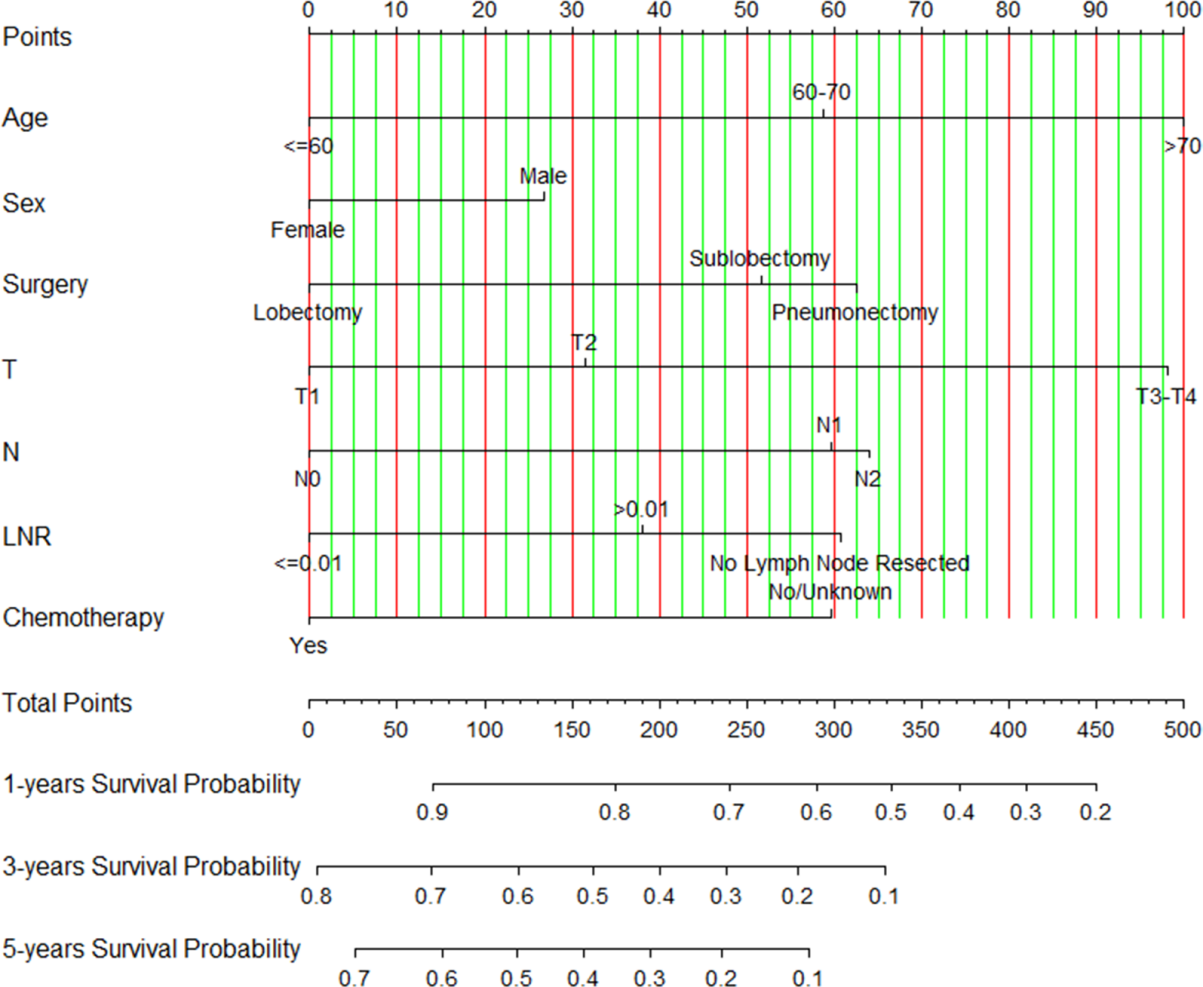
Nomograms to predict 1-, 3- and 5-year overall survival (OS) probability for resectable small-cell lung cancer (SCLC)

We built a nomogram based on above prognostic analyses for 1-, 3- and 5-year overall survival (Fig. 1). Each factor can obtain a corresponding point by drawing a line straight upward to the “Point axis”. Total point can be obtained by summing up the point of each factor, which can find a position on the “Total Points axis”. Then the predicting probability of 1-, 3- and 5-year OS can be got by drawing a line straight downward from the “Total Points axis” to corresponding “survival axis”. For example, a 65-year-old (58.75 points) female (0 point) received lobectomy (0 point) and adjuvant chemotherapy (0 point), who had T2 (31.50 points), N1 (59.75 points), and LNR > 0.01 (38 points). For this example, the total points equaled 188 score, and the suspected 1-year survival is approximately 78% (44% for 3-year survival and 32% for 5-year survival) (Additional file 4).

### Calibration and validation of the nomogram

There were 56 events (deaths) in the validation cohort and the mean follow-up period was 55.3 months (median, 42.5 months; range, 2–177 months).

**Fig. 2:**
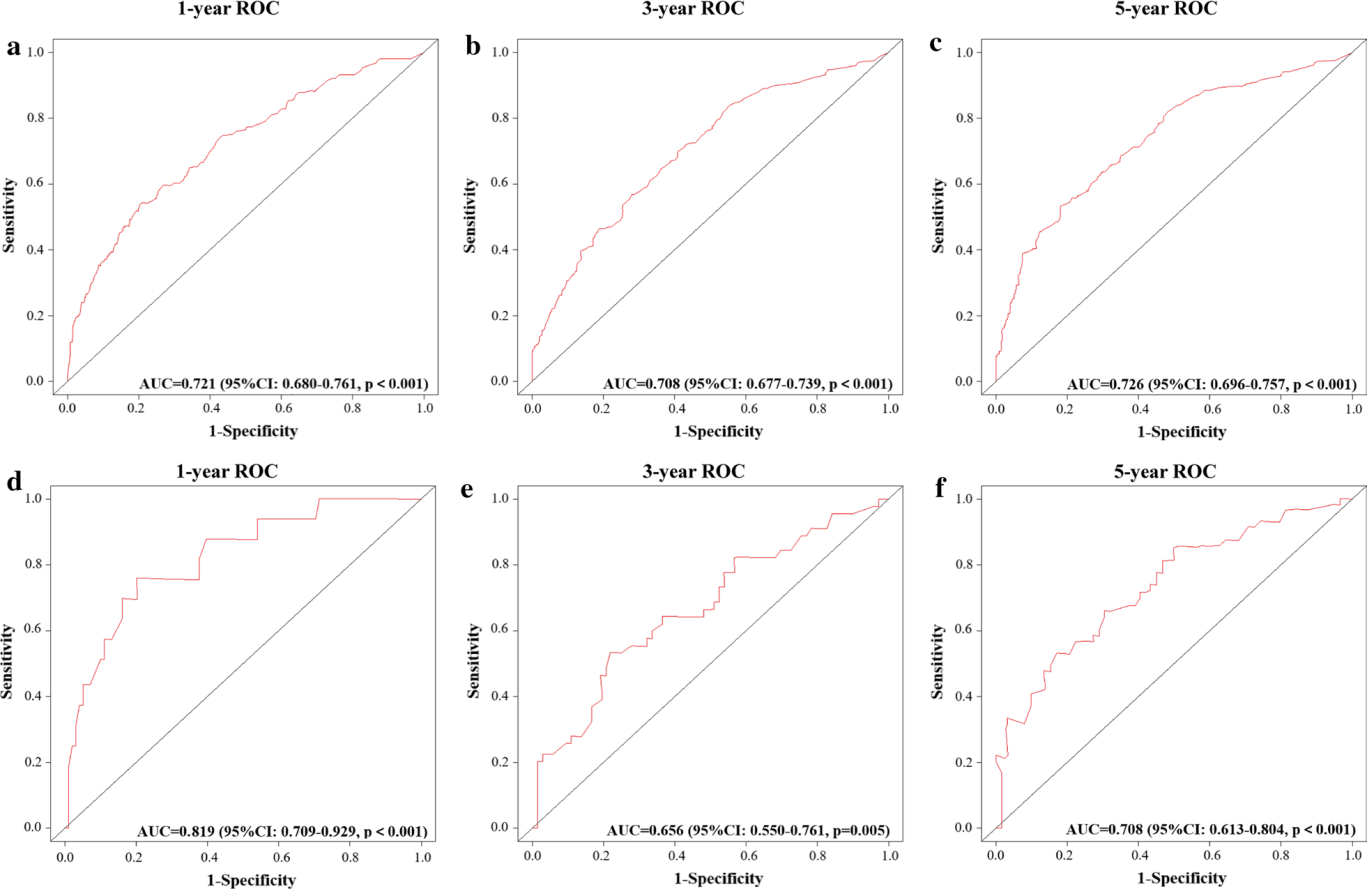
Validation of proposed nomogram by Receiver operating characteristic (ROC) analyses. **a**–**c** ROC curves for discrimination in the training cohort for 1-year (**a**), 3-year (**b**) and 5-year (**c**) overall survival. **d**–**f** ROC curves for discrimination in the validation cohort for 1-year (**d**), 3-year (**e**) and 5-year (**f**) overall survival. *AUC* area under the curve

**Fig. 3:**
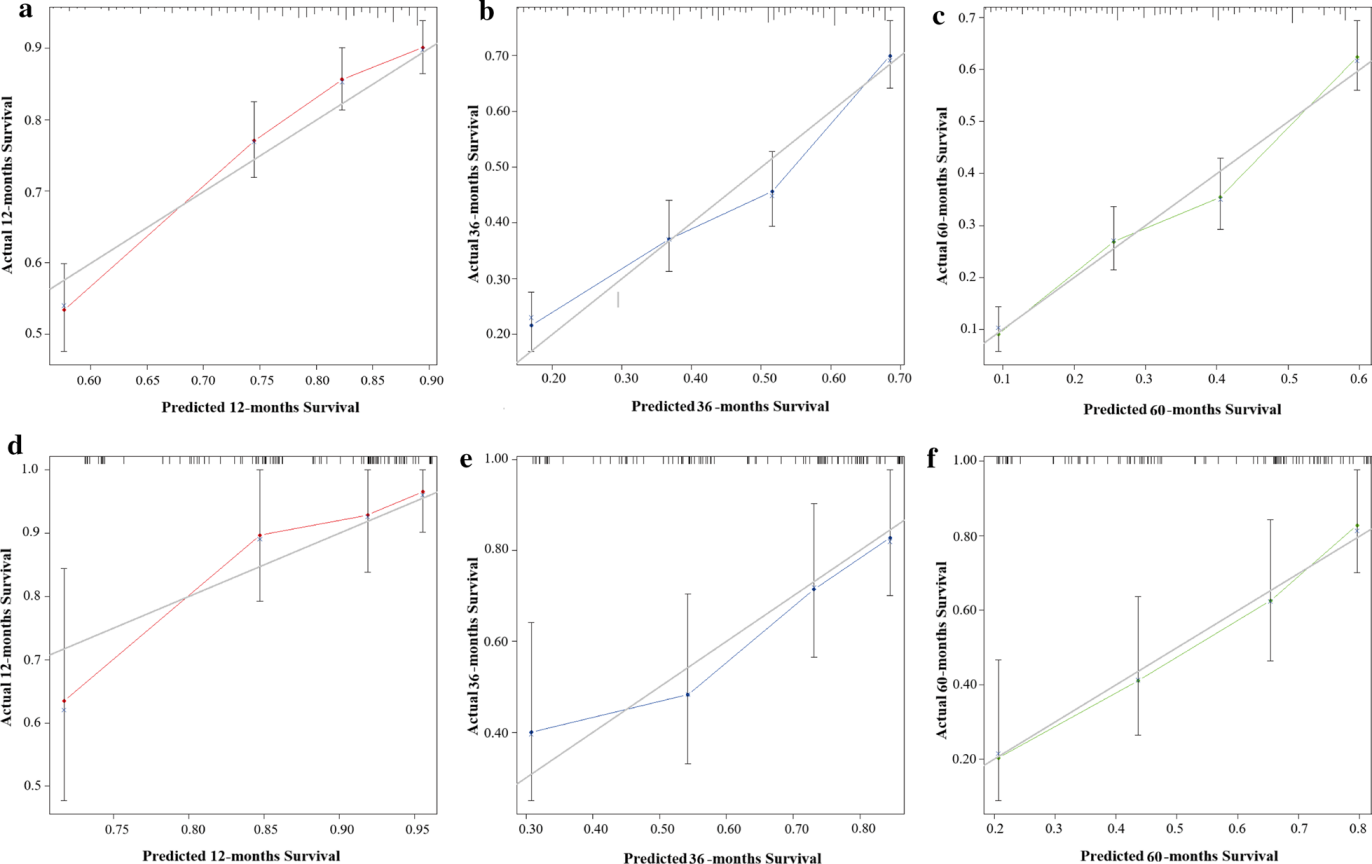
Validation of the nomogram by calibration curves. **a**–**c** The calibration curves of the model for **a** 1-year, **b** 3-year and 5-year **c** overall survival in the training cohort. **d**–**f** The calibration curves of the model for **d** 1-year, **e** 3-year and 5-year **f** overall survival in the validation cohort. Y-axis indicated the actual survival probability and x-axis indicated the predicated survival probability. The grey line indicated that prediction agrees with actuality. Error bars represent 95% confidence intervals

C-index of the training cohort were 0.721 (95% CI 0.680–0.761, p < 0.001), 0.708 (95% CI 0.677–0.739, p < 0.001), 0.726 (95% CI 0.696–0.757, p < 0.001) for 1-, 3- and 5-year OS, respectively. And that in the validation cohort were 0.819 (95% CI 0.709–0.929, p < 0.001), 0.656 (95% CI 0.550–0.761, p = 0.005), 0.708 (95% CI 0.599–0.793, p < 0.001) respectively. The data indicated brilliant discrimination ability of the nomogram (Fig. 2). The calibration curves were shown in Fig. 3. These curves presented an excellent consistency between predicted and actual survival conditions in the training cohort. Calibration curves of external validation cohort also showed an acceptable consistency between the model prediction and the actual observation for 1-, 3-, and 5-year OS.

### Comparison of the nomogram and 8th edition AJCC TNM staging system

**Fig. 4:**
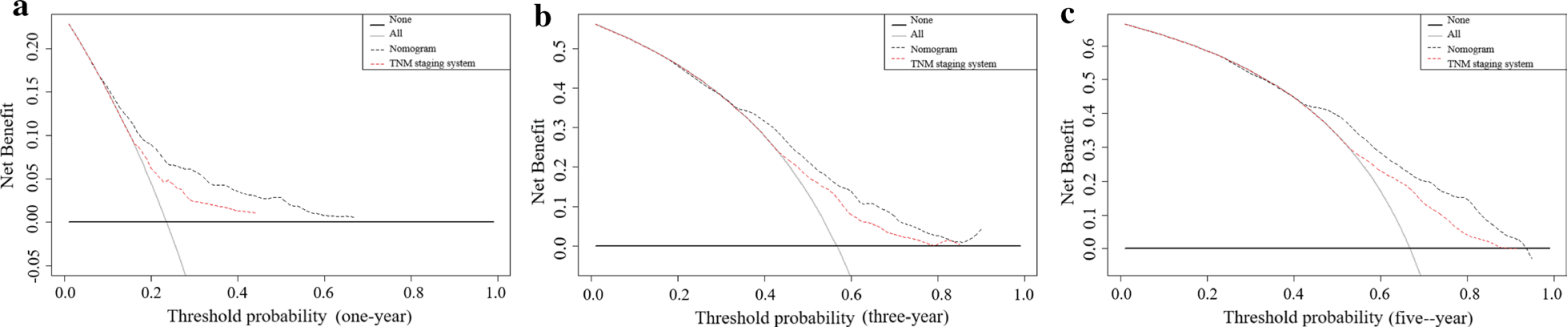
Decision curve analyses (DCA) of the nomogram and 8th edition AJCC TNM staging system for 1-year (**a**), 3-year (**b**), and 5-year (**c**) overall survival. The x-axis represents the threshold probabilities, and the y-axis measures the net benefit. The horizontal line along the x-axis assumes that overall death occurred in no patients, whereas the solid gray line assumes that all patients will have overall death at a specific threshold probability. The grey dashed line represents the nomogram. The red dashed line represents 8th edition AJCC TNM staging system

DCA analyses suggested significantly increased net benefits of the new nomogram over 8th edition AJCC TNM staging system with wide and practical ranges of threshold probabilities (Fig. 4). Above all, the nomogram can obtain more benefits in clinical application for predicting individual survival outcomes.

In the IDI analyses, the new nomogram performed better than TNM staging system. The 1-, 3- and 5-year IDI of the nomogram compared to TNM staging system was 5.0% (p < 0.001), 8.0% (p < 0.001) and 7.8% (p < 0.001), respectively (Additional file 2).

### Performance of the new risk stratification model

**Fig. 5:**
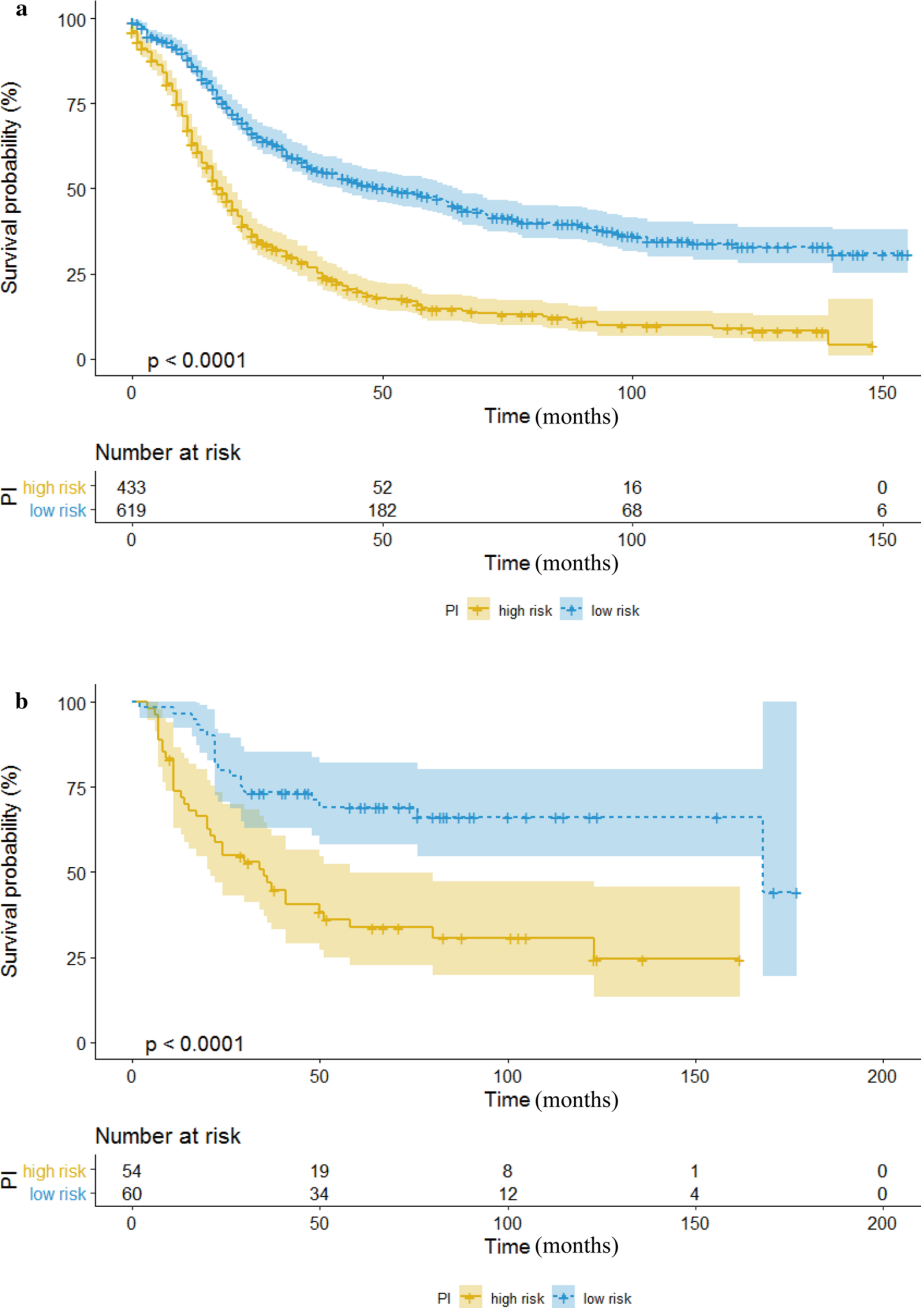
Kaplan–Meier survival analyses to test the risk stratification system within the training (**a**) and the validation cohort (**b**). The yellow line represents low-risk group, and the blue line represents high-risk group

The cutoff point of high-risk and low-risk cohort determined by ROC analysis was 202.355. And all 1052 patients in the training cohort were divided into high-risk group (Total points > 202.355) and low-risk group (Total points ≤ 202.355) based on this cutoff value. The 433 high-risk patients had significantly worse OS than the 619 low-risk patients (p < 0.0001) by Kaplan–Meier analyses (Fig. 5). Applying this cutoff value could also remarkably distinguish high-risk group from low-risk group in the validation cohort (p < 0.0001) (Fig. 5).

### Creation of a webserver for the nomogram

The public online version of our nomogram is available at https://prediction-tool.shinyapps.io/Nomogram-for-Resectable-SCLC/. Clinicians can use it very simply which doesn’t need any password.

## Discussion

SCLC is well recognized as an easily aggressive tumor which rarely amendable to surgical resection [2]. But surgery was demonstrated to increase local-control rate in certain early-stage SCLC [8, 9]. Besides, SCLC was definitely diagnosed until intraoperation occasionally, and resectable ones of which were considered to receive surgery [10]. Survival outcomes of resectable SCLC varies from patient to patient. Existent VALSG or TNM staging system are not efficient in predicting individualized OS of resectable SCLC patient. Therefore, we constructed and externally validated a clinical prognostic model that assign predictions for OS of resectable SCLC based on surgery and other clinicopathological variables. When applied to the external validation cohort, the new model achieved considerable discrimination ability and calibration accuracy (Figs. 2, 3). The C-index in the validation cohort were 0.819, 0.656, 0.708, respectively for 1-, 3- and 5-year OS. And further DCA and IDI analyses testified its obvious clinical application benefit versus TNM staging system. The risk stratification model according to this nomogram can effectively stratify patients in training or validation cohort into two risk groups (high-risk and low-risk) with distinguish OS. Besides, we provided a webserver to clinicians for more facile individual survival prediction.

By COX regression analyses, we identified age, sex, T stage, N stage, LNR, surgery and chemotherapy as independent predictors of overall survival. Some of these variables have been studied in previous research for their influence on survival of SCLC [12, 23–26]. Elder patients had worse survival than the younger ones might because degenerative changes in various aspects of organs function and increased prevalence of all types of comorbidities [27]. The male had worse survival than the female, which was consistent with the studies of Wang et al. and Xiao et al. [20, 21]. Lymph nodes metastatic ratio, as a new meaningful indicator for OS of SCLC, was also recognized as an independent predictor [22, 25]. The ones with lymph nodes resection performed had better survival than who not, which suggested to conduct lymphadenectomy for resectable SCLC. As with the nomogram of Wang et al. AJCC eighth TNM staging system contributed the most to the final risk score [21]. Notably, in addition to the common investigated factors, surgical procedure was a crucial independent predictor for OS, among which lobectomy posed the superior choice with better survival [8, 10, 13, 15].

There were a certain number of existing nomograms that involved SCLC patients in. However, most of these models were designed for pan-stage of the diagnosed SCLC, and no one of them has included specific surgical procedure in. Xie et al. built two nomograms by simply divided patients into limited or extensive stage but failed to involve information of tumor pathology like definite tumor size and lymph nodes status. In contrast, we brought in data of 8th edition AJCC T-stage and N-stage of every case to get a better understanding of the tumors. But the work of incorporating hematological markers into the models was worth emulating for our further study. Although these two models of Xie et al. received a considerable C-index (0.730) in internal validation, externally validation of them in a larger number of patients at multiple institutions should be considered [18]. Another single-institution study published by Xiao et al. built a nomogram to serve prediction of 3- and 5-year OS for SCLC. C-index of its nomogram (= 0.60) was not high enough might due to heterogeneity of the included patients which covered pan-stage SCLC, or due to lack of data of more detailed T, N, M information. What different from our study was that it didn’t regard surgery as a single factor in therapeutic regimen and didn’t mention the number of resected SCLC patients [20]. Pan et al. built a nomogram for SCLC that included only a small sample size of patients with resected SCLC (n = 53 in primary cohort, n = 4 in validation cohort) [19]. In 2018, a quality study constructed an update model based on a large sample size of cases and summarized previous three nomogram to a webserver. But its validation was performed by data of more recent years but from the same database as the training cohort, which would limit its generalizability. And it simply pointed out if surgery done or not, without mention of different surgical procedure, which means it might be not suitable for resectable SCLC patients [21]. In contrary to previous researches, our new model was constructed specially for resectable SCLC based on a large-population database and included common surgical procedure. Besides, the new model received ideal C-index by independent external validation which proved its better predicting accuracy.

Limitations have to be admitted in this study. Firstly, all of the data were obtained retrospectively, which made it susceptible to the inherent weaknesses of retrospective data collection. Although SEER is a huge population-based database, it doesn’t have data of tumor marker associated with SCLC such as NSE, proGRP and inflammation-related hematological markers both of which are key determinants of tumor survival [26]. The nomogram built by Xie et al. and Pan et al. both included such important information to efficiently increase model accuracy [18, 19]. What’s more, the chemotherapy regimens were unavailable and heterogeneous because this retrospective research collected data of different institutions over a long period. Finally, the sample size of our validation cohort was not very large. Another external validation with larger sample size of the predictive model is still necessary.

## Conclusions

Univariable and multivariable analyses identified seven baseline clinical and pathology characteristics that formed the basis of a nomogram to assist in predicting the OS of individual resectable SCLC patients. The choice of surgical procedure was identified as an important factor in the nomogram. This predicting tool consistently achieved appreciable predictive accuracy, reliability and clinical applicability by external validation. The use of this nomogram to distinguish between risk groups may aid in clinical decision-making for this patient population. Besides, we implemented this nomogram in a user-friendly webserver to clinicians and patients. This nomogram will be updated by including more meaningful factors in the future.

## Supplementary information

**Additional file 1.** Flow chart of this study.

**Additional file 2. **R running reports in this study.

**Additional file 3. **TRIPOD principle.

**Additional file 4. **Example of how to use this nomogram.

## Data Availability

The datasets used and analysed during the current study are available from the corresponding author on reasonable request.

## Abbreviations

SCLC: Small-cell lung cancer
C-index: Concordance index
DCAs: Decision curve analysis
ROC: The receiver operating characteristic analysis
IDI: Integrated discrimination improvement
HR: Hazard ratio
SEER database: The Surveillance, Epidemiology, and End Results
TRIPODL: Transparent Reporting of a multivariable prediction model for Individual Prognosis or Diagnosis
