# Supplementary material for: Development and validation of a prognostic model of resectable small-cell lung cancer: a large population-based cohort study and external validation
Source: J Transl Med. 2020 Jun 15;18:237. doi: 10.1186/s12967-020-02412-x (PMC7296644; doi:10.1186/s12967-020-02412-x)
Supplement: Supplementary file 1 — Additional file 1. Flow chart of this study. [file 12967_2020_2412_MOESM1_ESM.pdf]

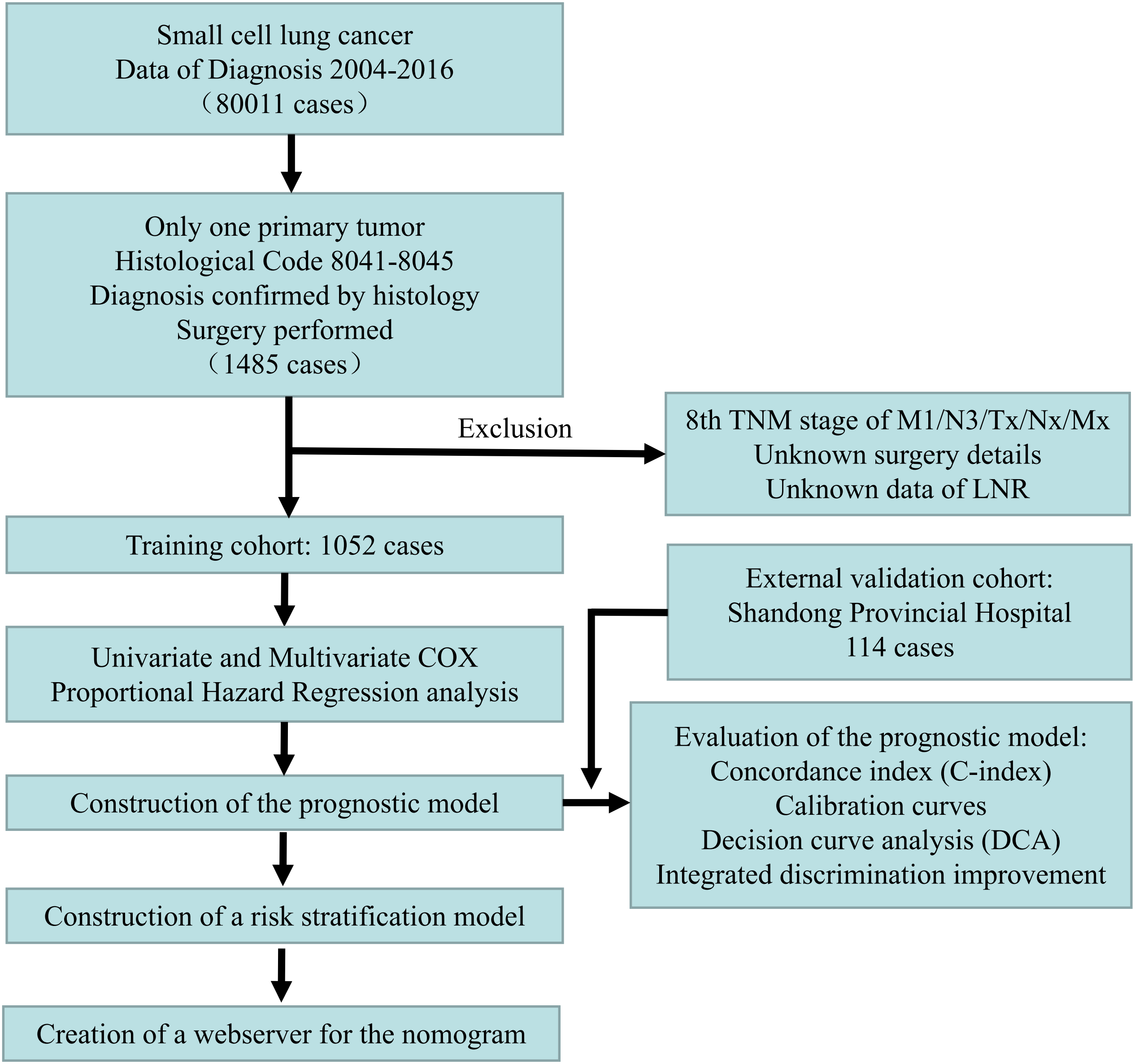
Small cell lung cancer
Data of Diagnosis 2004-2016
（80011 cases）
Only one primary tumor
Histological Code 8041-8045
Diagnosis confirmed by histology
Surgery performed
（1485 cases）
Exclusion
8th TNM stage of M1/N3/Tx/Nx/Mx
Unknown surgery details
Unknown data of LNR
Training cohort: 1052 cases
External validation cohort:
Shandong Provincial Hospital
114 cases
Univariate and Multivariate COX
Proportional Hazard Regression analysis
Evaluation of the prognostic model:
Concordance index (C-index)
Calibration curves
Decision curve analysis (DCA)
Integrated discrimination improvement
Construction of the prognostic model
Construction of a risk stratification model
Creation of a webserver for the nomogram
