# Supplementary material for: Development and validation of a prognostic model of resectable small-cell lung cancer: a large population-based cohort study and external validation
Source: J Transl Med. 2020 Jun 15;18:237. doi: 10.1186/s12967-020-02412-x (PMC7296644; doi:10.1186/s12967-020-02412-x)
Supplement: Supplementary file 2 — Additional file 2. R running reports in this study. [file 12967_2020_2412_MOESM2_ESM.pdf]

R running of Nomogram for Resected SCLC

This is the R running report of nomogram development and validation of the cohort using R software. (The IASLC validation was conducted by the biostatisticians using the same methods.) Text in blue represents the codes and text in blue represents the corresponding output.

1.R code for nomogram:

```
library(rms)
library(foreign)
library(survival)
setwd("C:/R work")

dev<-read.csv("dev.csv")
head(dev)
      id Sex Age T N Surgery LNR Chemotherapy follow death
1   1    1    2 1 1       1    1            2     46     1
2   3    2    2 2 2       1    2            1     20     1
3   4    2    2 3 2       3    2            1     17     1
4   5    2    3 1 1       1    1            2     10     1
5   6    1    2 3 1       1    1            1    103     1
6   7    2    2 2 3       2    2            1     20     1
str(dev)
'data.frame':   1052 obs. of   10 variables:
 $ id           : int  1 3 4 5 6 7 8 9 10 11 ...
 $ Sex          : int  1 2 2 2 1 2 1 2 2 2 ...
 $ Age          : int  2 2 2 3 2 2 3 1 3 3 ...
 $ T            : int  1 2 3 1 3 2 3 3 2 2 ...
 $ N            : int  1 2 2 1 1 3 1 3 1 1 ...
 $ Surgery      : int  1 1 3 1 1 2 2 2 1 1 ...
 $ LNR          : int  1 2 2 1 1 2 3 2 1 1 ...
 $ Chemotherapy: int  2 1 1 2 1 1 1 1 2 1 ...
 $ follow       : int  46 20 17 10 103 20 33 28 31 42 ...
 $ death        : int  1 1 1 1 1 1 1 1 1 1 ...
dev$Age<-factor(dev$Age,labels=c('<=60','60-70','>70'))
dev$Sex<-factor(dev$Sex,labels=c('Female','Male'))
dev$Surgery<-factor(dev$Surgery,labels=c('Lobectomy','Sublobectomy','Pneumonectomy'))
dev$T<-factor(dev$T,labels=c('T1','T2','T3-T4'))
dev$N<-factor(dev$N,labels=c('N0','N1','N2'))
dev$LNR<-factor(dev$LNR,labels=c('<=0.01','>0.01','No Lymph Node Resected'))
dev$Chemotherapy<-factor(dev$Chemotherapy,labels=c('Yes','No/Unknown'))

str(dev)
'data.frame':   1052 obs. of   10 variables:
 $ id           : int  1 3 4 5 6 7 8 9 10 11 ...
 $ Sex          : Factor w/ 2 levels "Female","Male": 1 2 2 2 1 2 1 2 2 2 ...
```

```
 $ Age           : Factor w/ 3 levels "<=60","60-70",..: 2 2 2 3 2 2 3 1 3 3 ...
 $ T             : int  1 2 3 1 3 2 3 3 2 2 ...
 $ N             : Factor w/ 3 levels "N0","N1","N2": 1 2 2 1 1 3 1 3 1 1 ...
 $ Surgery       : Factor w/ 3 levels "Lobectomy","Sublobar Ectomy",..: 1 1 3 1 1 2 2 2 1 1 ...
 $ LNR           : Factor w/ 3 levels "<=0.01",">0.01",..: 1 2 2 1 1 2 3 2 1 1 ...
 $ Chemotherapy: Factor w/ 2 levels "Yes","No/Unknown": 2 1 1 2 1 1 1 1 2 1 ...
 $ follow        : int  46 20 17 10 103 20 33 28 31 42 ...
 $ death         : int  1 1 1 1 1 1 1 1 1 1 ...
y<-Surv(dev$follow,dev$death==1,type="right")
mod<-coxph(y~Age + Sex + Surgery + T + N + LNR + Chemotherapy, data=dev)
temp <- cox.zph(mod,transform="rank",global=F)
temp

                                   rho    chisq        p
Age60-70                      -0.03277   0.7162 3.97e-01
Age>70                        -0.02537   0.4176 5.18e-01
SexMale                        0.00537   0.0190 8.90e-01
SurgerySublobar Ectomy         0.01548   0.1736 6.77e-01
SurgeryPneumonectomy          -0.02646   0.4635 4.96e-01
T                             -0.08378   4.8307 2.80e-02
NN1                           -0.03090   0.6058 4.36e-01
NN2                           -0.05470   1.9656 1.61e-01
LNR>0.01                       0.01310   0.1104 7.40e-01
LNRNo Lymph Node Detected -0.01071   0.0829 7.73e-01
ChemotherapyNo/Unknown        -0.24712 40.1353 2.37e-10

ddist <- datadist(dev)
options(datadist='ddist')

units(dev$follow) <- "Month"

fcox <- cph(Surv(follow,death) ~ Age + Sex + Surgery + T + N + LNR + Chemotherapy,surv=T,x=T, y=T,data=dev)

surv <- Survival(fcox)
nom <- nomogram(fcox, fun=list(function(x) surv(12, x),
                               function(x) surv(36, x),
                               function(x) surv(60, x)),
                funlabel=c("1-years Survival Probability",
                           "3-years Survival Probability",
                           "5-years Survival Probability"),lp=F)
plot(nom)
```

```
surv <- Survival(fcox)
nom <- nomogram(fcox, fun=list(function(x) surv(12, x),
                               function(x) surv(36, x),
                               function(x) surv(60, x)),
                funlabel=c("1-years Survival Probability",
                           "3-years Survival Probability",
                           "5-years Survival Probability"),lp=F)
plot(nom,col.grid=c("red","green","blue"))
```

2.R code for ROC curve:

```
library(rms)
library(foreign)
library(survival)

setwd("C:/R work")
source("stdca.R")
dev<-read.csv("dev.csv")
head(dev)
str(dev)

dev$Age<-factor(dev$Age,labels=c('<=60','60-70','>70'))
dev$Sex<-factor(dev$Sex,labels=c('Female','Male'))
dev$Surgery<-factor(dev$Surgery,labels=c('Lobectomy','Sublobectomy','Pneumonectomy'))
dev$T<-factor(dev$T,labels=c('T1','T2','T3-T4'))
dev$N<-factor(dev$N,labels=c('N0','N1','N2'))
dev$LNR<-factor(dev$LNR,labels=c('<=0.01','>0.01','No Lymph Node Resected'))
dev$Chemotherapy<-factor(dev$Chemotherapy,labels=c('Yes','No/Unknown'))

str(dev)

library(survivalROC)
nobs<-NROW(dev)
cutoff1<-12
cutoff2<-36
Cutoff3<-60

Srv=Surv(dev$follow,dev$death)

coxmod=coxph(Srv ~   Age + Sex + Surgery + T + N + LNR + Chemotherapy,data=dev)

summary(coxmod)
Call:
coxph(formula = Srv ~ Age + Sex + Surgery + T + N + LNR + Chemotherapy,
    data = dev)
```

```
n= 1052, number of events= 650

                              coef exp(coef) se(coef)      z Pr(>|z|)
Age60-70                   0.42254   1.52584  0.10529 4.013 5.99e-05 ***
Age>70                     0.71858   2.05152  0.10546 6.814 9.50e-12 ***
SexMale                    0.19270   1.21252  0.07943 2.426 0.015272 *
SurgerySublobectomy        0.37066   1.44869  0.09719 3.814 0.000137 ***
SurgeryPneumonectomy       0.45003   1.56836  0.19603 2.296 0.021688 *
TT2                        0.22629   1.25394  0.09228 2.452 0.014196 *
TT3-T4                     0.70473   2.02330  0.10704 6.584 4.59e-11 ***
NN1                        0.42896   1.53566  0.18466 2.323 0.020182 *
NN2                        0.46019   1.58438  0.16449 2.798 0.005148 **
LNR>0.01                   0.27311   1.31404  0.17979 1.519 0.128752
LNRNo Lymph Node Resected 0.43604    1.54657  0.13237 3.294 0.000987 ***
ChemotherapyNo/Unknown     0.42872   1.53529  0.08883 4.826 1.39e-06 ***
---
Signif. codes:  0 ‘***’ 0.001 ‘**’ 0.01 ‘*’ 0.05 ‘.’ 0.1 ‘ ’ 1

                          exp(coef) exp(-coef) lower .95 upper .95
Age60-70                      1.526     0.6554    1.2413     1.876
Age>70                        2.052     0.4874    1.6684     2.523
SexMale                       1.213     0.8247    1.0377     1.417
SurgerySublobectomy           1.449     0.6903    1.1974     1.753
SurgeryPneumonectomy          1.568     0.6376    1.0680     2.303
TT2                           1.254     0.7975    1.0465     1.503
TT3-T4                        2.023     0.4942    1.6404     2.496
NN1                           1.536     0.6512    1.0693     2.205
NN2                           1.584     0.6312    1.1477     2.187
LNR>0.01                      1.314     0.7610    0.9238     1.869
LNRNo Lymph Node Resected     1.547     0.6466    1.1932     2.005
ChemotherapyNo/Unknown        1.535     0.6513    1.2900     1.827

Concordance= 0.669  (se = 0.012 )
Likelihood ratio test= 212.1  on 12 df,   p=<2e-16
Wald test            = 208.2  on 12 df,   p=<2e-16
Score (logrank) test = 217.1  on 12 df,   p=<2e-16
```

```
Sexn<- as.numeric(dev$Sex)
dev$sexpoint<- ifelse(Sexn==1,0,0.19270)

Agen<- as.numeric(dev$Age)
dev$agepoint<- ifelse(Agen==1,0,ifelse(Agen==2,0.42254,0.71858))
```

```
Surgeryn<- as.numeric(dev$Surgery)
dev$surgerypoint<- ifelse(Surgeryn==1,0,ifelse(Surgeryn==2,0.37066,0.45003))

Tn<- as.numeric(dev$T)
dev$tpoint<- ifelse(Tn==1,0,ifelse(Tn==2,0.22629,0.70473))

Nn<- as.numeric(dev$N)
dev$npoint<- ifelse(Nn==1,0,ifelse(Nn==2,0.42896,0.46019))

LNRn<- as.numeric(dev$LNR)
dev$lnrpoint<- ifelse(LNRn==1,0,ifelse(LNRn==2,0.27311,0.43604))

Chemotherapyn<- as.numeric(dev$Chemotherapy)
dev$chemotherapypoint<- ifelse(Chemotherapyn==1,0,0.42872)

dev$PI<-dev$points<-rowSums(dev[,c("agepoint","sexpoint","surgerypoint","lnrpoint","tpoint","npoint","chemotherapypoint")])

data<-dev[which(dev$death!="NA"),]

write.csv(dev, "devnew.csv")

SROC= survivalROC(Stime = data$follow, status = data$death, marker = data$PI, predict.time =cutoff1, method= "KM" )
cut.op= SROC$cut.values[which.max(SROC$TP-SROC$FP)]
cut.op
[1] 1.68211
plot(SROC$FP,SROC$TP, type="l", xlim=c(0,1), ylim=c(0,1),
     xlab = paste( "FP","\n", "AUC = ",round(SROC$AUC,3)),
     ylab = "TP", col="red")
abline(0,1)
legend("bottomright",c("One Year Survival Probability PI"),col="red",lty=c(1,1))

SROC= survivalROC(Stime = data$follow, status = data$death, marker = data$PI, predict.time =cutoff2, method= "KM" )
cut.op= SROC$cut.values[which.max(SROC$TP-SROC$FP)]
cut.op
[1] 1.1521
plot(SROC$FP,SROC$TP, type="l", xlim=c(0,1), ylim=c(0,1),
     xlab = paste( "FP","\n", "AUC = ",round(SROC$AUC,3)),
     ylab = "TP", col="red")
abline(0,1)
legend("bottomright",c("Three Year Survival Probability PI "),col="red",lty=c(1,1))
```

```
SROC= survivalROC(Stime = data$follow, status = data$death, marker = data$PI, predict.time
=cutoff3, method= "KM" )
cut.op= SROC$cut.values[which.max(SROC$TP-SROC$FP)]
cut.op
[1] 1.43803
plot(SROC$FP,SROC$TP, type="l", xlim=c(0,1), ylim=c(0,1),
     xlab = paste( "FP","\n", "AUC = ",round(SROC$AUC,3)),
     ylab = "TP", col="red")
abline(0,1)
legend("bottomright",c("Five Year Survival Probability PI "),col="red",lty=c(1,1))


library(rms)
library(foreign)
library(survival)

setwd("C:/R work")
source("stdca.R")
vad<-read.csv("vad.csv")
head(vad)
str(vad)

vad$Age<-factor(vad$Age,labels=c('<=60','60-70','>70'))
vad$Sex<-factor(vad$Sex,labels=c('Female','Male'))
vad$Surgery<-factor(vad$Surgery,labels=c('Lobectomy','Sublobectomy','Pneumonectomy'))
vad$T<-factor(vad$T,labels=c('T1','T2','T3-T4'))
vad$N<-factor(vad$N,labels=c('N0','N1','N2'))
vad$LNR<-factor(vad$LNR,labels=c('<=0.01','>0.01','No Lymph Node Resected'))
vad$Chemotherapy<-factor(vad$Chemotherapy,labels=c('Yes','No/Unknown'))

str(vad)

library(survivalROC)
nobs<-NROW(vad)
cutoff1<-12
cutoff2<-36
Cutoff3<-60

Srv=Surv(vad$follow,vad$death)

coxmod=coxph(Srv ~   Age + Sex + Surgery + T + N + LNR + Chemotherapy,data=vad)

Sexn<- as.numeric(vad$Sex)
vad$sexpoint<- ifelse(Sexn==1,0,0.19270)
```

```
Agen<- as.numeric(vad$Age)
vad$agepoint<- ifelse(Agen==1,0,ifelse(Agen==2,0.42254,0.71858))

Surgeryn<- as.numeric(vad$Surgery)
vad$surgerypoint<- ifelse(Surgeryn==1,0,ifelse(Surgeryn==2,0.37066,0.45003))

Tn<- as.numeric(vad$T)
vad$tpoint<- ifelse(Tn==1,0,ifelse(Tn==2,0.22629,0.70473))

Nn<- as.numeric(vad$N)
vad$npoint<- ifelse(Nn==1,0,ifelse(Nn==2,0.42896,0.46019))

LNRn<- as.numeric(vad$LNR)
vad$lnrpoint<- ifelse(LNRn==1,0,ifelse(LNRn==2,0.27311,0.43604))

Chemotherapyn<- as.numeric(vad$Chemotherapy)
vad$chemotherapypoint<- ifelse(Chemotherapyn==1,0,0.42872)

vad$PI<-vad$points<-rowSums(vad[,c("agepoint","sexpoint","surgerypoint","lnrpoint","tpoint","n
point","chemotherapypoint")])

data<-vad[which(vad$death!="NA"),]

write.csv(vad, "vadnew.csv")

SROC= survivalROC(Stime = data$follow, status = data$death, marker = data$PI, predict.time
=cutoff1, method= "KM" )
cut.op= SROC$cut.values[which.max(SROC$TP-SROC$FP)]
cut.op
[1] 1.7835
plot(SROC$FP,SROC$TP, type="l", xlim=c(0,1), ylim=c(0,1),
     xlab = paste( "FP","\n", "AUC = ",round(SROC$AUC,3)),
     ylab = "TP", col="red")
abline(0,1)
legend("bottomright",c("One Year Survival Probability PI"),col="red",lty=c(1,1))

SROC= survivalROC(Stime = data$follow, status = data$death, marker = data$PI, predict.time
=cutoff2, method= "KM" )
cut.op= SROC$cut.values[which.max(SROC$TP-SROC$FP)]
cut.op
[1] 1.61335
plot(SROC$FP,SROC$TP, type="l", xlim=c(0,1), ylim=c(0,1),
     xlab = paste( "FP","\n", "AUC = ",round(SROC$AUC,3)),
```

```
        ylab = "TP", col="red")
abline(0,1)
legend("bottomright",c("Three Year Survival Probability PI "),col="red",lty=c(1,1))

SROC= survivalROC(Stime = data$follow, status = data$death, marker = data$PI, predict.time
=cutoff3, method= "KM" )
cut.op= SROC$cut.values[which.max(SROC$TP-SROC$FP)]
cut.op
[1] 1.61335
plot(SROC$FP,SROC$TP, type="l", xlim=c(0,1), ylim=c(0,1),
        xlab = paste( "FP","\n", "AUC = ",round(SROC$AUC,3)),
        ylab = "TP", col="red")
abline(0,1)
legend("bottomright",c("Five Year Survival Probability PI "),col="red",lty=c(1,1))
```

3.R code for calibration plot:

```
library(rms)
library(foreign)
library(survival)
setwd("C:/R  work")

dev<-read.csv("dev.csv")
head(dev)
str(dev)

dev$Age<-factor(dev$Age,labels=c('<=60','60-70','>70'))
dev$Sex<-factor(dev$Sex,labels=c('Female','Male'))
dev$Surgery<-factor(dev$Surgery,labels=c('Lobectomy','Sublobectomy','Pneumonectomy'))
dev$T<-factor(dev$T,labels=c('T1','T2','T3-T4'))
dev$N<-factor(dev$N,labels=c('N0','N1','N2'))
dev$LNR<-factor(dev$LNR,labels=c('<=0.01','>0.01','No Lymph Node Resected'))
dev$Chemotherapy<-factor(dev$Chemotherapy,labels=c('Yes','No/Unknown'))

str(dev)

ddist  <-  datadist(dev)
options(datadist='ddist')

units(dev$follow)  <-  "Month"
fcox  <-  cph(Surv(follow,death)  ~  Age  +  Sex  +  Surgery  +  T  +  N  +  LNR  +  Chemotherapy,surv
=T,x=T,  y=T,data=dev)

fcox1  <-  cph(Surv(follow,death)  ~Age  +  Sex  +  Surgery  +  T  +  N  +  LNR  +  Chemotherapy,surv
=T,x=T,  y=T,time.inc  =  12,data=dev)
```

```
cal1 <- calibrate(fcox1, cmethod="KM", method="boot", u=12, m=250, B=1000)

plot(cal1,col=c("red"))

fcox2 <- cph(Surv(follow,death) ~Age + Sex + Surgery + T + N + LNR + Chemotherapy,surv
=T,x=T, y=T,time.inc = 36,data=dev)

cal2 <- calibrate(fcox2, cmethod="KM", method="boot", u=36, m=250, B=1000)

plot(cal2,col=c("blue"))

fcox2 <- cph(Surv(follow,death) ~Age + Sex + Surgery + T + N + LNR + Chemotherapy,surv
=T,x=T, y=T,time.inc = 60,data=dev)

cal2 <- calibrate(fcox2, cmethod="KM", method="boot", u=60, m=250, B=1000)

plot(cal2,col=c("green"))


vad<-read.csv("vad.csv")

vad$Age<-factor(vad$Age,labels=c('<=60','60-70','>70'))
vad$Sex<-factor(vad$Sex,labels=c('Female','Male'))
vad$Surgery<-factor(vad$Surgery,labels=c('Lobectomy','Sublobectomy','Pneumonectomy'))
vad$T<-factor(vad$T,labels=c('T1','T2','T3-T4'))
vad$N<-factor(vad$N,labels=c('N0','N1','N2'))
vad$LNR<-factor(vad$LNR,labels=c('<=0.01','>0.01','No Lymph Node Resected'))
vad$Chemotherapy<-factor(vad$Chemotherapy,labels=c('Yes','No/Unknown'))

str(vad)

ddist <- datadist(vad)
options(datadist='ddist')

units(vad$follow) <- "Month"

fcox <- cph(Surv(follow,death) ~ Age + Sex + Surgery + T + N + LNR + Chemotherapy,sur
v=T,x=T, y=T,data=vad)

fvad1 <- cph(Surv(follow, death) ~predict(fcox, newdata=vad), x=T, y=T, surv=T, data=vad,
time.inc=12)
cfvad1 <- calibrate(fvad1, cmethod="KM", method="boot", u=12, m=26, B=1000)
plot(cfvad1,col=c("red"))
```

```
fvad2 <- cph(Surv(follow, death) ~predict(fcox, newdata=vad), x=T, y=T, surv=T, data=vad,
time.inc=36)
cfvad2 <- calibrate(fvad2, cmethod="KM", method="boot", u=36, m=26, B=1000)
plot(cfvad2,col=c("blue"))

fvad2 <- cph(Surv(follow, death) ~predict(fcox, newdata=vad), x=T, y=T, surv=T, data=vad,
time.inc=60)
cfvad2 <- calibrate(fvad2, cmethod="KM", method="boot", u=60, m=26, B=1000)
plot(cfvad2,col=c("green"))
```

4. R code for DCA curve:

```
library(rms)
library(foreign)
library(survival)
setwd("C:/R work")
dev<-read.csv("dev.csv")
head(dev)
str(dev)

dev$Age<-factor(dev$Age,labels=c('<=60','61-70','>70'))
dev$Sex<-factor(dev$Sex,labels=c('Female','Male'))
dev$Surgery<-factor(dev$Surgery,labels=c('Lobectomy','Sublobectomy','Pneumonectomy'))
dev$T<-factor(dev$T,labels=c('T1','T2','T3-T4'))
dev$N<-factor(dev$N,labels=c('N0','N1','N2'))
dev$LNR<-factor(dev$LNR,labels=c('<=0.01','>0.01','No Lymph Node Resected'))
dev$Chemotherapy<-factor(dev$Chemotherapy,labels=c('Yes','No/Unknown'))

str(dev)

ddist <- datadist(dev)
options(datadist='ddist')
units(dev$os) <- "Month"

y<-Surv(dev$os,dev$ status ==1,type="right")
mod1<-coxph(y~ Age + Sex + Surgery + T + N + LNR + Chemotherapy, data=dev)
summary(mod1)

fcox1 <- cph(Surv(os, status) ~ Age + Sex + Surgery + T + N + LNR + Chemotherapy,surv=T,x=T,
y=T,data=dev)
med    <- Quantile(fcox1)
nom.sur1 <- nomogram(fcox1, fun=function(x) med(lp=x), funlabel="Median Survival
Time",lp=F)
plot(nom.sur1)
```

```
surv <- Survival(fcox1)
nom1 <- nomogram(fcox1, fun=list(function(x) surv(12, x),
                                 function(x) surv(36, x),
                                 function(x) surv(60, x)),
                 funlabel=c("1-years Survival Probability",
                            "3-years Survival Probability",
                            "5-years Survival Probability"),lp=F)
plot(nom1)

library(nomogramEx)
nomogramEx(nomo=nom1, np=3, digit=7)
$RESULT
[1] "The equation of each variable as follows:"

[[2]]
          Age
1     0.00000
2    58.80252
3   100.00000

[[3]]
        Sex
4   0.00000
5  26.81631

[[4]]
    Surgery
6   0.00000
7  51.58236
8  62.62947

[[5]]
           T
9     0.00000
10   31.49101
11   98.07385

[[6]]
           N
12    0.00000
13   59.69837
14   64.04490
```

```
[[7]]
          LNR
15   0.00000
16  38.00325
17  60.68040

[[8]]
   Chemotherapy
18          0.00000
19         59.66232

[[9]]
[1] "1-years Survival Probability = 0 * points ^3 + -6.5e-06 * points ^2 + 0.0004805 * points + 0.8956765"

[[10]]
[1] "3-years Survival Probability = 0 * points ^3 + -8.3e-06 * points ^2 + -0.0009098 * points + 0.8024442"

[[11]]
[1] "5-years Survival Probability = 0 * points ^3 + -8.9e-06 * points ^2 + -0.0012152 * points + 0.7362696"
```

```
dev$Agepoint<- ifelse(dev$Age=="<=60",0,ifelse(dev$Age=="61-70", 58.80252,100))
dev$Surgerypoint<- ifelse(dev$Surgery=="Lobectomy",0,ifelse(dev$Surgery=="Sublobectomy", 51.58236,62.62947))
dev$Sexpoint<- ifelse(dev$Sex=="Female",0, 26.81631)
dev$Tpoint<- ifelse(dev$T=="T1",0,ifelse(dev$T=="T2", 31.49101,98.07385))
dev$Npoint<- ifelse(dev$N=="N0",0,ifelse(dev$N=="N1", 59.69837,64.04490))
dev$LNRpoint<- ifelse(dev$LNR=="<=0.01",0,ifelse(dev$LNR==">0.01", 38.00325,60.68040))
dev$Chemotherapypoint<- ifelse(dev$Chemotherapy=="Yes",0, 59.66232)
dev$points1 <- dev$Agepoint + dev$Surgerypoint + dev$Sexpoint + dev$Tpoint + dev$Npoint + dev$LNRpoint + dev$Chemotherapypoint

dev$one.years.Survival.Probabilitynew1 = c(summary(survfit(mod1,newdata=dev),times=12)$surv)

dev$one.years.death.Probabilitynew1 =1- dev$one.years.Survival.Probabilitynew1

dev$three.years.Survival.Probabilitynew1 = c(summary(survfit(mod1,newdata=dev),times=36)$surv)

dev$three.years.death.Probabilitynew1 =1- dev$three.years.Survival.Probabilitynew1
```

```
dev$five.years.Survival.Probabilitynew1 = c(summary(survfit(mod1,newdata=dev),times=60)$s
urv)

dev$five.years.death.Probabilitynew1 =1- dev$five.years.Survival.Probabilitynew1


y<-Surv(dev$os,dev$ status ==1,type="right")
mod2<-coxph(y~ T + N , data=dev)
summary(mod2)

fcox2 <- cph(Surv(os, status) ~T+N,surv=T,x=T, y=T,data=dev)
med  <- Quantile(fcox2)

nom.sur2 <- nomogram(fcox2, fun=function(x) med(lp=x), funlabel="Median Survival Time",
lp=F)
plot(nom.sur2)

surv <- Survival(fcox2)
nom2 <- nomogram(fcox2, fun=list(function(x) surv(12, x),
                                  function(x) surv(36, x),
                                  function(x) surv(60, x)),
                     funlabel=c("1-years Survival Probability",
                                "3-years Survival Probability",
                                "5-years Survival Probability"),lp=F)
plot(nom2)

library(nomogramEx)
nomogramEx(nomo=nom2, np=3, digit=2)
$RESULT
[1] "The equation of each variable as follows:"

[[2]]
  T
1 0
2 0
3 0

[[3]]
    N
4   0
5   0
6 100

[[4]]
```

```
[1] "1-years Survival Probability = 0 * points ^3 + 0 * points ^2 + 0 * points + 0.89"

[[5]]
[1] "3-years Survival Probability = 0 * points ^3 + 0 * points ^2 + 0 * points + 0.71"

[[6]]
[1] "5-years Survival Probability = 0 * points ^3 + 0 * points ^2 + 0 * points + 0.65"
```

```
dev$Tpoint<-  ifelse(dev$T=="T1",0,ifelse(dev$T=="T2",  0,0))
dev$Npoint<-  ifelse(dev$N=="N0",0,ifelse(dev$N=="N1",  0,100))
dev$points2  <-  dev$Tpoint  +  dev$Npoint

dev$one.years.Survival.Probabilitynew2  =  c(summary(survfit(mod2,newdata=dev),times=12)$surv)

dev$one.years.death.Probabilitynew2 =1- dev$one.years.Survival.Probabilitynew2

dev$three.years.Survival.Probabilitynew2  =  c(summary(survfit(mod2,newdata=dev),times=36)$surv)

dev$three.years.death.Probabilitynew2 =1- dev$three.years.Survival.Probabilitynew2

dev$five.years.Survival.Probabilitynew2  =  c(summary(survfit(mod2,newdata=dev),times=60)$surv)

dev$five.years.death.Probabilitynew2 =1- dev$five.years.Survival.Probabilitynew2

source("stdca.R")

stdca(data=dev,outcome="status",ttoutcome="os",timepoint=12,
predictors=c("one.years.death.Probabilitynew1",
"one.years.death.Probabilitynew2") ,smooth=TRUE ,probability=c("TRUE", "TRUE"))
stdca(data=dev,outcome="status",ttoutcome="os",timepoint=36,
predictors=c("three.years.death.Probabilitynew1",
"three.years.death.Probabilitynew2") ,smooth=TRUE ,probability=c("TRUE", "TRUE"))
stdca(data=dev,outcome="status",ttoutcome="os",timepoint=60,
predictors=c("five.years.death.Probabilitynew1",
"five.years.death.Probabilitynew2") ,smooth=TRUE ,probability=c("TRUE", "TRUE"))

stdca(data=dev,outcome="status",ttoutcome="os",timepoint=12,                predictors=c("points1",
"points2") ,smooth=TRUE ,probability=c("FALSE", "FALSE"))
```

5.R code for K-M curve and logrank test

```
library(rms)
library(foreign)
library(survival)
library(nricens)
setwd("C:/R work")
devnew<-read.csv("devnew.csv")
head(devnew)
str(devnew)
library("survival")
fit <- survfit(Surv(follow,death)
~ PI, data = devnew)
class(fit)
library("survminer")
ggsurvplot(fit, data = devnew)

ggsurvplot(fit, data = devnew, fun = "event")
ggsurvplot(fit, data = devnew, fun = "cumhaz")

ggsurvplot(fit, data = devnew, conf.int = TRUE, pval = TRUE, fun = "pct", risk.table = TRUE, size = 1, linetype = "strata", palette = c("#E7B800", "#2E9FDF"), legend = "bottom",legend.title = "PI",legend.labs = c("high risk","low risk"))

library(rms)
library(foreign)
library(survival)
library(nricens)
setwd("C:/R work")
vadnew<-read.csv("vadnew.csv")
head(vadnew)
str(vadnew)
library("survival")
fit <- survfit(Surv(follow,death)
~ PI, data = vadnew)
class(fit)
library("survminer")
ggsurvplot(fit, data = vadnew)

ggsurvplot(fit, data = vadnew, fun = "event")
ggsurvplot(fit, data = vadnew, fun = "cumhaz")

ggsurvplot(fit, data = vadnew, conf.int = TRUE, pval = TRUE, fun = "pct", risk.table = TRUE, size = 1, linetype = "strata", palette = c("#E7B800", "#2E9FDF"), legend = "bottom",legend.title = "PI",legend.labs = c("high risk","low risk"))
```

6.R code for IDI

```
library(rms)
library(foreign)
library(survival)
library(nricens)

setwd("C:/R work")

dev<-read.csv("dev.csv")
head(dev)
str(dev)

dev$Age<-factor(dev$Age,labels=c('<=60','60-70','>70'))
dev$Sex<-factor(dev$Sex,labels=c('Female','Male'))
dev$Surgery<-factor(dev$Surgery,labels=c('Lobectomy','Sublobectomy','Pneumonectomy'))
dev$T<-factor(dev$T,labels=c('T1','T2','T3-T4'))
dev$N<-factor(dev$N,labels=c('N0','N1','N2'))
dev$LNR<-factor(dev$LNR,labels=c('<=0.01','>0.01','No Lymph Node Resected'))
dev$Chemotherapy<-factor(dev$Chemotherapy,labels=c('Yes','No/Unknown'))

str(dev)

mstd=coxph(Surv(dev$follow,dev$death==1)~T + N,x=TRUE,data=dev)

mnew=coxph(Surv(dev$follow,dev$death==1)~ Age + Sex + Surgery + T + N + LNR +
Chemotherapy,x=TRUE,data=dev)

Srv=Surv(dev$follow,dev$death)

dev$oldone.years.Survival.Probability = c(1-
(summary(survfit(mstd,newdata=dev),times=12)$surv))
dev$newone.years.Survival.Probability = c(1-
(summary(survfit(mnew,newdata=dev),times=12)$surv))

devevent<-dev[dev$death==1,]
devnonevent<-dev[dev$death==0,]

IDIevent<- devevent$newone.years.Survival.Probability -
devevent$oldone.years.Survival.Probability
IDInonevent<- devnonevent$newone.years.Survival.Probability -
devnonevent$oldone.years.Survival.Probability

mean(IDIevent)
[1] 0.02302352
mean(IDInonevent)
```

```
[1] -0.02718314
IDI= mean(IDIevent)- mean(IDInonevent)
IDI
[1] 0.05020666
SEevent = sd(IDIevent)/sqrt(length(IDIevent))
SEnonevnt = sd(IDInonevent)/sqrt(length(IDInonevent))

Z = IDI/sqrt(SEevent*SEevent + SEnonevnt*SEnonevnt )
Z
[1] 9.204749
P= 1- pnorm(Z)
P
[1] 0
dev$oldthree.years.Survival.Probability = c(1-(summary(survfit(mstd,newdata=dev),times=36)$surv))
dev$newthree.years.Survival.Probability = c(1-(summary(survfit(mnew,newdata=dev),times=36)$surv))

devevent<-dev[dev$death==1,]
devnonevent<-dev[dev$death==0,]

IDIevent<- devevent$newthree.years.Survival.Probability - devevent$oldthree.years.Survival.Probability
IDInonevent<- devnonevent$newthree.years.Survival.Probability - devnonevent$oldthree.years.Survival.Probability

mean(IDIevent)
[1] 0.0292221
mean(IDInonevent)
[1] -0.05058821
IDI= mean(IDIevent)- mean(IDInonevent)
IDI
[1] 0.07981032
SEevent = sd(IDIevent)/sqrt(length(IDIevent))
SEnonevnt = sd(IDInonevent)/sqrt(length(IDInonevent))

Z = IDI/sqrt(SEevent*SEevent + SEnonevnt*SEnonevnt )
Z
[1] 8.894716
P= 1- pnorm(Z)
P
[1] 0
dev$oldfive.years.Survival.Probability = c(1-(summary(survfit(mstd,newdata=dev),times=60)$surv))
```

```
dev$newfive.years.Survival.Probability = c(1-
(summary(survfit(mnew,newdata=dev),times=60)$surv))

devevent<-dev[dev$death==1,]
devnonevent<-dev[dev$death==0,]

IDIevent<- devevent$newfive.years.Survival.Probability -
devevent$oldfive.years.Survival.Probability
IDInonevent<- devnonevent$newfive.years.Survival.Probability -
devnonevent$oldfive.years.Survival.Probability

mean(IDIevent)
[2] 0.02560333
mean(IDInonevent)
[2] -0.05282392
IDI= mean(IDIevent)- mean(IDInonevent)
IDI
[2] 0.07842724
SEevent = sd(IDIevent)/sqrt(length(IDIevent))
SEnonevnt = sd(IDInonevent)/sqrt(length(IDInonevent))

Z = IDI/sqrt(SEevent*SEevent + SEnonevnt*SEnonevnt )
Z
[2] 8.604544
P= 1- pnorm(Z)
P
[1] 0
```

Year	IDI	p value
One-year	0.050	< 0.001
Three-year	0.080	< 0.001
Five-year	0.078	< 0.001
