# Supplementary figures and images for: Development and validation of a prognostic model of resectable small-cell lung cancer: a large population-based cohort study and external validation
Source: J Transl Med. 2020 Jun 15;18:237. doi: 10.1186/s12967-020-02412-x (PMC7296644; doi:10.1186/s12967-020-02412-x)

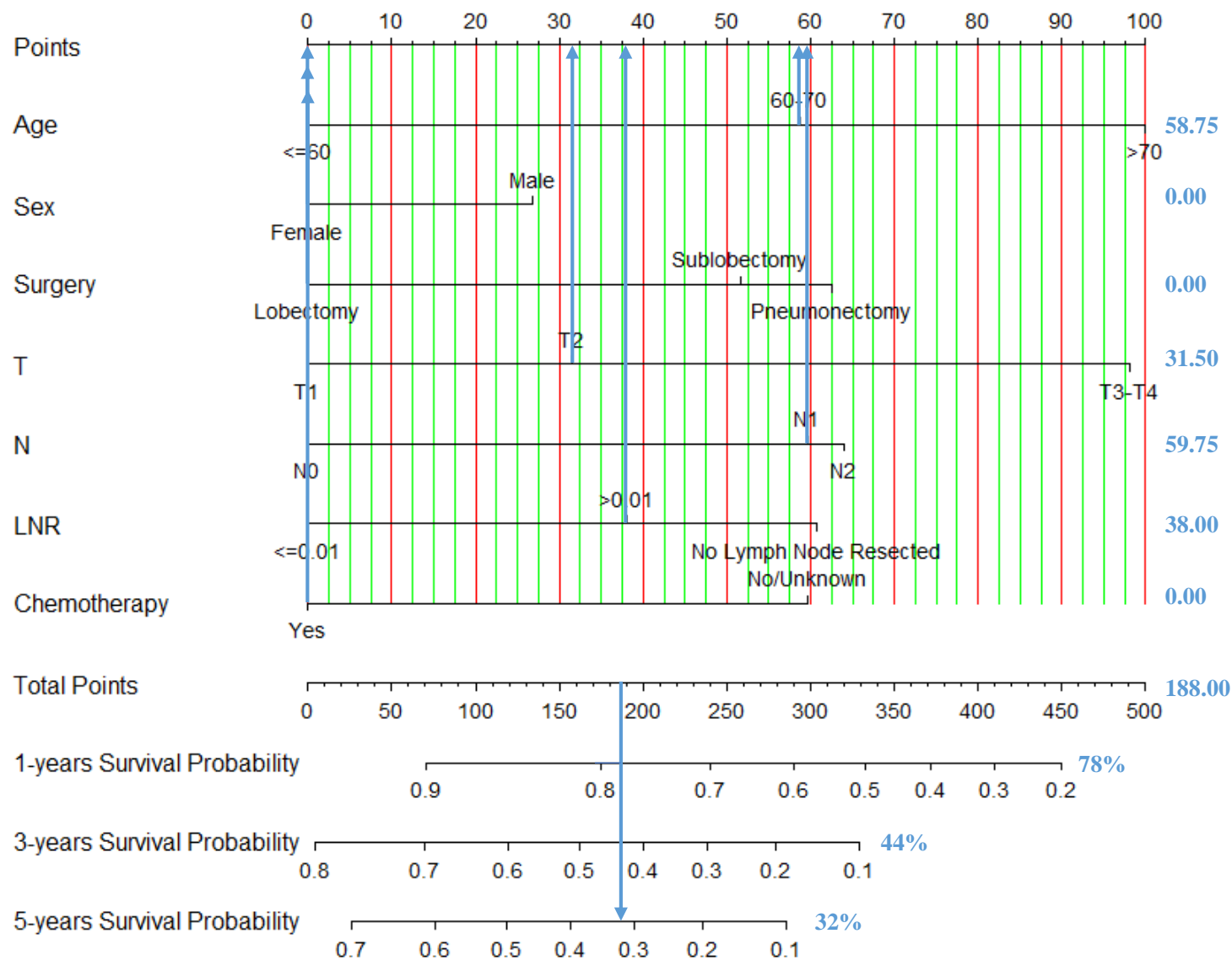

Supplement: Supplementary file 4 — Additional file 4. Example of how to use this nomogram. [file 12967_2020_2412_MOESM4_ESM.pdf]
